# Antiprotozoal Activities of Organic Extracts from French Marine Seaweeds

**DOI:** 10.3390/md9060922

**Published:** 2011-05-25

**Authors:** Catherine Vonthron-Sénécheau, Marcel Kaiser, Isabelle Devambez, Antoine Vastel, Isabelle Mussio, Anne-Marie Rusig

**Affiliations:** 1 Pharmacognosie et Molécules Naturelles Bio-actives, Laboratoire d’Innovation Thérapeutique UMR CNRS 7200, Faculté de Pharmacie, Université de Strasbourg, 74 route du Rhin, 67401 Illkirch cedex, France; 2 Physiologie et Ecophysiologie des Mollusques Marins, UMR M IFREMER 100, Institut de Biologie Fondamentale et Appliquée (IBFA), Université de Caen Basse-Normandie, 14032 Caen cedex, France; E-Mails: devambez@unicaen.fr (I.D.); vastarel35@hotmail.fr (A.V.); isabelle.mussio@unicaen.fr (I.M.); anne-marie.rusig@unicaen.fr (A.-M.R.); 3 Swiss Tropical and Public Health Institution, Socinstrasse 57, 4002 Basel, Switzerland; E-Mail: Marcel.Kaiser@unibas.ch; 4 University of Basel, Petersplatz 1, 4003 Basel, Switzerland

**Keywords:** seaweeds, Phaeophyceae, Rhodophyceae, *Plasmodium*, *Trypanosoma*, *Leishmania*

## Abstract

Marine macrophytes contain a variety of biologically active compounds, some reported to have antiprotozoal activity *in vitro*. As a part of a screening program to search for new natural antiprotozoals, we screened hydroalcoholic and ethyl acetate extracts of 20 species of seaweeds from three phyla (Rhodophyta, Heterokontophyta and Chlorophyta), sampled along the Normandy (France) coast. We tested them *in vitro* against the protozoa responsible for three major endemic parasitic diseases: *Plasmodium falciparum*, *Leishmania donovani* and *Trypanosoma cruzi*. The selectivity of the extracts was also evaluated by testing on a mammalian cell line (L6 cells). Ethyl acetate extracts were more active than hydroalcoholic ones. Activity against *T. cruzi* and *L. donovani* was non-existent to average, but almost half the extracts showed good activity against *P. falciparum*. The ethyl acetate extract of *Mastocarpus stellatus* showed the best antiplasmodial activity as well as the best selectivity index (IC_50_ = 2.8 μg/mL; SI > 30). Interestingly, a red algae species, which shares phylogenetic origins with *P. falciparum*, showed the best antiplasmodial activity. This study is the first to report comparative antiprotozoal activity of French marine algae. Some of the species studied here have not previously been biologically evaluated.

## 1. Introduction

Protozoal diseases, especially malaria, leishmaniasis, and Chagas disease, are major causes of mortality in various tropical and subtropical regions [1]. Moreover, treatment failure is increasingly common with current drugs. The resistance of *Plasmodium falciparum,* for example, to the former first-line antimalarials, chloroquine and sulfadoxine/pyrimethamine, has reached critical levels in many malaria-endemic regions and is producing deleterious effects on human health, wealth, and lifespans. Recent reports that malaria parasites have begun to develop tolerance to the artemisinin-based combination therapies threaten the last mainstay for treating uncomplicated malaria in endemic countries [2,3]. Furthermore, no new class of antimalarials has been introduced into clinical practice since 1996 [4]. Worse, chemotherapy of visceral leishmaniasis and Chagas disease still relies on first-line drugs that are old and far from satisfactory because of their major side effects, limited efficacy, and difficulty of administration. New more effective chemotherapeutic agents with novel modes of action are needed, and marine biodiversity might be a source of chemodiversity for that purpose.

Marine biodiversity contains resources composed of a variety of biologically active compounds [5–7]. Marine macrophytes produce an array of secondary compounds [8] still underexploited for their biomedical potential although they are a particularly available biomass. Studies of tropical marine macrophytes have shown their extensive biological activity, including antiprotozoal [9–12], but very little is known about Western seaweed species.

As a part of a screening program to search for new natural antiprotozoal products, we report here the *in vitro* screening of 35 polar (hydroalcoholic) and apolar (ethyl acetate) extracts from 20 species of seaweeds from the Normandy (France) coast against cultured protozoa responsible for human malaria, visceral leishmaniasis, and Chagas disease.

## 2. Results and Discussion

The sampling resulted in the selection of 20 species of seaweeds that were brown (8), red (9) and green algae (3). The species were collected from rocky habitats along the Normandy coast in northern France (Table 1).

The potential antiplasmodial activity of the resultant ethyl acetate and hydroalcoholic extracts was evaluated *in vitro* against erythrocytes infected by a resistant K1 strain of *P. falciparum*, as well as against *T. cruzi* trypomastigotes and *Leishmania donovani* amastigotes (Table 2). Extracts were first screened at two concentrations (1.6 and 9.7 μg/mL), and parasite growth inhibition was measured. Extracts for which parasite growth inhibition was greater than 50% at the concentration of 9.7 μg/mL were subsequently assayed to determine their IC_50_. An extract was considered as active if the IC_50_ value was less than 5 μg/mL. Cytotoxicity to primary mammalian L6 cells was also evaluated to determine the selectivity of its activity. Table 3 presents the IC_50_ values and selectivity indexes (ratio of cytotoxic to antiprotozoal activity).

The active extracts were almost entirely (97%) ethyl acetate extracts, while the hydroalcoholic extracts were mainly inactive. This finding suggests that the active antiprotozoal compounds were relatively apolar, except for the hydroalcoholic extract of *C. crispus*, which was quite active against *L. donovani* (95% inhibition of parasite growth at 9.7 μg/mL).

**Figure 1:**
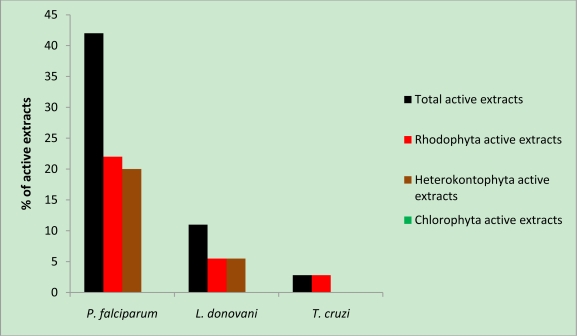
Percentage of extracts active against the protozoan parasites tested.

*P. falciparum* was the pathogen most responsive to these extracts: 40% of the extracts showed activity against this protozoon. *L. donovani* was less sensitive (11% of the extracts were active against it) and *T. cruzi* quite insensitive (3%). Red and brown seaweeds were almost equally active against *P. falciparum* and *L. donovani,* but green seaweeds were inactive (Figure 1).

Few studies have reported antiprotozoal screening of marine algae, and those that did looked at tropical and Asian species [13,14], most often individually [5,6]. Trypanocidal and leishmanicidal activity was recently assessed in British and Irish species [15,16]. Those studies found that organic extracts from several British green algae species were active, although weakly, against the same strains of *T. cruzi* and *L. donovani* that we used here [16]. *U. lactuca*, the only green algae common to their study and ours, was inactive in ours at 9.7 μg/mL, despite similar experimental conditions. This discrepancy may stem either from the different extraction solvents or from their different phytochemical compositions, probably due to the different habitats of the two samples.

Brown tropical algae have previously been shown to be moderately active against *L. mexicana* promastigotes, while red and green algal organic extracts were almost inactive, as in our study [14]. Here, the most active extract against *L. donovani* axenic amastigotes was the ethyl acetate extract of *B. bifurcata,* which had an IC_50_ value of 3.9 μg/mL. Nevertheless, the selectivity index of 1.6 found for this extract seems to indicate general toxicity. Moreover, crude organic extracts of Irish *B. bifurcata* were recently shown to be active against both *L. donovani* and *T. brucei rhodesiense*, the protozoan parasite responsible for sleeping sickness [15]. *B. bifurcata* contains arrays of terpenoids [17–19] with cytotoxic activity [19,20]. The ethyl acetate that we used here efficiently extracts this type of compound. Their presence could explain the absence of selectivity.

Our results provide further evidence that marine seaweeds may be active against protozoa, especially *Plasmodium* species. Investigations of red algae species are rare, still more so against *P. falciparum*. We showed here, for the first time, *in vitro* antiplasmodial activity by eight French species of red algae: *C. jubata, C. crispus, D. carnosa, G. latifolium, G. gracilis, G. turuturu, H. flosculosus, and M. stellatus*. Moreover, the IC_50_ values measured for these extracts reflect activity similar to that reported for such reference extracts as ethanolic crude extracts of *Artemisia annua* (Quinguao) and of *Azadirachta indica* (Neem) in the same *in vitro* microdilution test under similar experimental conditions [21,22].

Interestingly, *Plasmodium* parasites contain a cellular peculiarity, called the apicoplast, a rudimentary plastid that was acquired long ago by secondary endosymbiosis between a free-living ancestor of Apicomplexans and a red algal species [23]. Accordingly, this organelle probably shares several metabolic pathways and housekeeping processes with red algae. We can hypothesize that some algal compounds contained in these extracts kill malaria parasites by interfering with these common pathways or processes.

The most active and interesting extract was the ethyl acetate extract of *M. stellatus,* which had the best IC_50_ and SI values (2.8 μg/mL and >30, respectively). Its selectivity towards *P. falciparum* was also good, compared with the other protozoan parasites we tested. To our knowledge, no antiplasmodial activity has previously been shown for *Mastocarpus* species. Moreover, phytochemical data for these genera are sparse. Nevertheless, some tropical species of red algae have been shown to contain such antiplasmodial compounds as halogenated diterpenes [6] or bromophycolides [5,24]. Bioguided fractionation associated with dereplication techniques should allow us to identify new antiplasmodial compounds from this species and thus help us to determine its mode of action.

## 3. Experimental Section

### 3.1. Algae Collection and Identification

The 20 algae species were collected between November 2005 and September 2007 at several locations on the coast of Basse-Normandie. Table 1 reports the collection dates and sites.

Taxonomic determination was performed by Dr. A.-M. Rusig and voucher specimens of the algae are deposited in the Herbarium of the University of Caen.

### 3.2. Preparation of Crude Extracts

Freeze-dried algal material of each species (200 g) was powdered and stirred overnight at room temperature for complete extraction, with 70% EtOH or ethyl acetate (10%, w/v). The filtrates were dried under vacuum at 35 °C and the residues were stored at 4 °C until testing. Tannins were removed from the crude hydroalcoholic extracts with Sephadex LH-20 exclusion chromatography, according to the method described by Houghton and Raman [25].

### 3.3. *In Vitro* Antiprotozoal Assays

The extracts were dissolved in dimethylsulfoxide (DMSO) to obtain a concentration of 10 mg/mL and screened for antiprotozoal activity against *P. falciparum*, *T. cruzi* and *L. donovani* and cytotoxicity against rat skeletal muscle myoblasts (L-6 cells). The *in vitro* assays were conducted as described by Scala *et al.* [26]. A brief description is given below.

#### 3.3.1. Activity against *P. Falciparum*

*In vitro* activity against erythrocytic stages of *P. falciparum* was determined by a modified [^3^H]-hypoxanthine incorporation assay with the chloroquine- and pyrimethamine-resistant K1 strain [27]. Briefly, parasite cultures incubated in RPMI 1640 medium with 5% Albumax (without hypoxanthine) were exposed to serial drug dilutions in microtiter plates. After 48 h of incubation at 37 °C in a reduced oxygen atmosphere, 0.5 μCi [^3^H]-hypoxanthine was added to each well. Cultures were incubated for a further 24 h before they were harvested onto glass-fiber filters and washed with distilled water. The radioactivity was counted with a BetaplateTM liquid scintillation counter (Wallac, Zurich, Switzerland). The results were recorded as counts per minute (CPM) per well at each drug concentration and expressed as the percentage of untreated controls. IC_50_ values were calculated from graphically plotted dose-response curves by linear interpolation. Chloroquine (Sigma C6628) and artemisinin (Sigma 36,159-3) were used as positive references.

#### 3.3.2. Activity against *Trypanosoma cruzi*

Rat skeletal myoblasts (L6 cells) were seeded in 96-well microtiter plates at 2000 cells/well in 100 μL RPMI 1640 medium with 10% FBS and 2 mM l-glutamine. After 24 h the medium was removed and replaced by 100 μL per well containing 5000 trypomastigote forms of *T. cruzi* Tulahuen strain C2C4 with the β-galactosidase (Lac Z) gene [28]. After 48 h, the medium was removed from the wells and replaced by 100 μL fresh medium with or without a serial drug dilution of seven 3-fold dilution steps covering a range from 90 to 0.123 μg/mL. After 96 h of incubation the plates were inspected under an inverted microscope to assure growth of the controls and sterility. Then the substrate CPRG/Nonidet (50 μL) was added to all wells. A color reaction developed within 2–6 h and could be read photometrically at 540 nm. The IC_50_ values were calculated from the sigmoidal inhibition curves with SoftmaxPro software. Benznidazole (Roche) was used as a positive reference.

#### 3.3.3. Activity against *Leishmania Donovani*

Amastigotes of *L. donovani* strain MHOM/ET/67/L82 were grown in axenic culture at 37 °C in SM medium at pH 5.4 supplemented with 10% heat-inactivated fetal bovine serum under an atmosphere of 5% CO_2_ in air. Culture medium (100 L) was seeded in 96-well microtiter plates with 10^5^ amastigotes from axenic culture with or without a serial drug dilution. Serial drug dilutions covering a range from 90 to 0.123 μg/mL were prepared. After 72 h of incubation the plates were inspected under an inverted microscope to assure growth of the controls and sterile conditions. Then 10 μL of a resazurin solution (12.5 mg resazurin dissolved in 100 mL double-distilled water) was added to each well, and the plates incubated for another 2 h. They were then read in a Spectramax Gemini XS microplate fluorometer at an excitation wavelength of 536 nm and an emission wavelength of 588 nm. The IC_50_ values were calculated from the sigmoidal inhibition curves with SoftmaxPro software. Miltefosin (Zentaris GmbH, Germany) was used as a positive reference.

### 3.4. Cytotoxicity against L6 cells

Assays were performed in 96-well microtiter plates, each well containing 100 L of RPMI 1640 medium supplemented with 1% l-glutamine (200 mM) and 10% fetal bovine serum, and 4 × 10^4^ l-6 cells (rat skeletal myoblasts). Serial drug dilutions of seven 3-fold dilution steps covering a range from 90 to 0.123 μg/mL were prepared. After 72 h of incubation the plates were inspected under an inverted microscope to assure growth of the controls and sterile conditions. Then 10 μL of a resazurin solution (12.5 mg resazurin dissolved in 100 mL distilled water) was added to each well and the plates incubated for another 2 h. They were then read with a Spectramax Gemini XS microplate fluorometer at an excitation wavelength of 536 nm and an emission wavelength of 588 nm. The IC_50_ values were calculated from the sigmoidal inhibition curves with SoftmaxPro software. Podophyllotoxin (Sigma, P4405) was used as a positive reference.

### 3.5. Calculation of IC_50_

To measure antiplasmodial activity, the concentration of extract at which the parasite growth (=[^3^H]hypoxanthine uptake) was inhibited by 50% (IC_50_) was calculated by linear interpolation between the two concentrations above and below 50% [29]. To assess leishmanicidal, antitrypanosomal, and cytotoxic activity we transferred data into the graphic Softmax Pro program (Molecular Devices), which calculated IC_50_ values from the sigmoidal inhibition curve. The values given in Table 3 are the means of two independent assays.

### 3.6. Selectivity Index Determination

The selectivity index (SI) corresponds to the ratio of the IC_50_ value of the cytotoxic activity to the IC_50_ value of antiprotozoal activity. An SI value >10 is generally considered to indicate antiprotozoal activity not due to general cytotoxicity.

## 4. Conclusions

Almost all the marine macrophytes species tested in the current study showed activity against at least one protozoan. Some of them may be promising sources for further bio-guided isolation of new active components. Most of these components are probably apolar compounds. The most interesting species, in terms of antiplasmodial activity and selectivity, is the red algae *M. stellatus*. Bioguided fractionation is underway.

## Figures and Tables

**Table 1:** Marine algal species selected for the study, and the sites and times of their collection (Normandy coast, France).

**Species**	**Family**	**Collection site**	**Collection time**
**Chlorophyta**			
*Codium tomentosum* Stackhouse	Codiaceae	Cap Lévy *(Manche)*	June 2007
*Ulva lactuca* (Linnaeus)	Ulvaceae	Luc-sur-Mer *(Calvados)*	October 2006
*Ulva clathrata* (Roth) C. Agardh	Ulvaceae	Anse St Martin *(Manche)*	June 2007
**Heterokontophyta**			
*Bifurcaria bifurcata* R. Ross	Sargassaceae	Cap Lévy *(Manche)*	June 2007
*Dictyopteris polypodioides* (A.P. de Candolle)	Dictyotaceae	Barneville *(Calvados)*	October 2007
J.V. Lamouroux			June 2007
*Dictyota dichotoma* (Hudson) J.V. Lamouroux	Dictyotaceae	Anse St Martin *(Manche)*	June 2007
*Fucus serratus* (Linnaeus)	Fucaceae	Luc-sur-mer *(Calvados)*	November 2005
*Himanthalia elongata* (Linnaeus)	Himanthaliaceae	Cap Lévy *(Manche)*	June 2006
*Laminaria digitata* (Linnaeus) J.V. Lamouroux	Laminariaceae	Langrunes-sur-Mer *(Calvados)*	January 2007
*Pelvetia canaliculata* Decaisne & Thuret	Fucaceae	Cap Lévy *(Manche)*	June 2006
*Sargassum muticum* (Yendo) Fensholt	Sargassaceae	Cap Lévy *(Manche)*	June 2006
**Rhodophyta**			
*Calliblepharis jubata* (Goodenough & woodward) Kützing	Cystocloniaceae	Cap Lévy *(Manche)*	June 2007
*Chondrus crispus* Stackhouse	Gigartinaceae	Cap Lévy *(Manche)*	June 2007
*Dilsea carnosa* (Schmidel) Kuntze	Dumontiaceae	Langrune-sur-Mer *(Calvados)*	January 2007
*Gelidium latifolium* Bornet ex Hauck	Gelidiaceae	Cap Lévy *(Manche)*	June 2006
*Gracilaria gracilis* (Stackhouse) Steentoft, L.M. Irvine & Farnham	Gracilariaceae	Anse St Martin *(Manche)*	June 2007
*Grateloupia turuturu* Yamada	Halymeniaceae	St Vaast-la-Hougue *(Manche)*	September 2007
*Halurus flosculosus* (J. Ellis) Maggs & Hommersand	Ceramiaceae	Anse St Martin *(Manche)*	June 2007
*Mastocarpus stellatus* (Stackhouse) Guiry	Phyllophoraceae	Cap Lévy *(Manche)*	June 2006
*Palmaria palmata* (Linnaeus) Kuntze	Palmariaceae	Luc-sur-Mer *(Calvados)*	November 2005

**Table 2:** *In vitro* antiprotozoal medium throughput screening of extracts obtained from the selected species. *P. falciparum*: Multidrug-resistant K1 strain erythrocytic stages; *T. cruzi*: Talahuen strain trypomastigotes; *L. donovani*: MHOM/ET/67/L82 strain axenic amastigotes.

		**Parasite growth inhibition (%)**					
		***P. falciparum Erythrocytic stages***		***T. cruzi Trypomastigotes***		***L. donovani Axenic amastigotes***	
**Species**	**Extract**	1.6 μg/mL	9.7μg/mL	1.6 μg/mL	9.7 μg/mL	1.6 μg/mL	9.7 μg/mL
*B. bifurcata*	E	0	25	18	0	16	40
	A	36	100	0	78	31	100
*C. jubata*	E	0	0	11	0	13	34
	A	3	71	19	0	20	40
*C. tomentosum*	E	0	2	0	0	15	29
	A	0	0	0	0	0	0
*C. crispus*	E	0	0	0	0	13	95
	A	28	92	1	0	0	12
*D. polypodioides*	E	nd					
	A	8	81	6	7	14	41
*D. dichotoma*	E	nd					
	A	19	98	21	17	27	83
*D. carnosa*	E	0	6	0	0	0	15
	A	9	71	10	14	9	31
*F. serratus*	E	0	0	0	0	0	15
	A	0	42	0	3	0	14
*G. latifolium*	E	nd					
	A	1	93	0	0	18	49
*G. gracilis*	E	0	0	0	9	21	29
	A	5	92	0	0	19	36
*G. turuturu*	E	nd					
	A	42	97	15	14	9	33
*H. flosculosus*	E	nd					
	A	19	94	17	21	22	49
*H. elongata*	E	0	5	17	13	20	40
	A	14	81	0	18	13	43
*L. digitata*	E	0	0	0	9	0	11
	A	0	64	0	3	0	8
*M. stellatus*	E	0	0	0	4	11	20
	A	42	94	8	0	22	39
*P. palmata*	E	0	0	0	0	0	0
	A	0	1	17	36	0	10
*P. canaliculata*	E	15	30	0	0	7	32
	A	0	41	0	13	14	37
*S. muticum*	E	0	0	0	1	8	37
	A	50	96	0	9	4	48
*U. lactuca*	E	0	0	0	3	0	26
	A	0	28	0	0	0	11
*U. clathrata*	E	0	0	0	0	0	0
	A	0	13	0	0	0	7

***Standard drugs***		0.003 μg/mL	0.018 μg/mL	0.5 μg/mL	2.4 μg/mL	0.2 μg/mL	1.2 μg/mL

Artemisinin		68	100				
Benznidazole				45	91		
Miltefosine						59	85

(**E**): EtOH 60% extract, (**A**): ethyl acetate extract, **nd**: not determined. Compounds for which parasite growth inhibition was greater than 50% at 9.7 μg/mL were assayed to determine IC_50_ and evaluate cytotoxicity.

**Table 3:** *In vitro* antiprotozoal and cytotoxic activities of the active ethyl acetate extracts. Data shown are means of two independent assays.

	**IC_50_ (μg/mL)**			**Selectivity index (SI)**
	**Antiprotozoal activity**		**Cytotoxic activity**	
**Species**	*P. falciparum*	*L. donovani*	L6 cells	
		
*B. bifurcata*	>5	3.8	6	1.6 ^b^
*C. jubata*	5	nd	71	14 ^a^
*C. crispus*	2.9	nd	84	29 ^a^
*D. polypodioides*	nd	10.8	87	8 ^b^
*D. dichotoma*	3.1	8.8	27	9 ^a^
*D. carnosa*	3.9	9.5	74	19 ^a^
*G. latifolium*	3.4	nd	62	18 ^a^
*G. gracilis*	3.3	nd	71	21 ^a^
*G. turuturu*	3.1	nd	71	23 ^a^
*H. flosculosus*	4.6	nd	58	12 ^a^
*H. elongata*	3.5	nd	88	25 ^a^
*M. stellatus*	2.8	nd	>90	>30 ^a^
*P. canaliculata*	nd	nd	87	11 ^a^
*S. muticum*	2.9	nd	63	11 ^a^
***Standards***				
Chloroquine	0.069	-	-	-
Miltefosine	-	0.181	-	-
Podophyllotoxin	-	-	0.007	-

**SI**: selectivity index, ratio of cytotoxic activity on L6 cells to the best antiprotozoal activity measured, that is, to antiplasmodial (SI^a^) or leishmanicidal (SI^b^) activity; **nd**: not determined.
